# Community engagement in global health education supports equity and advances local priorities: an eight year Ecuador-Canada partnership

**Published:** 2018-05-31

**Authors:** Shivali Misra, Alison Doucet, Juana Morales, Neil Andersson, Ann Macaulay, Andrea Evans

**Affiliations:** 1 Department of Family Medicine, McGill University, Quebec, Canada; 2 Chilcapamba, Canton of Cotacachi, Ecuador; 3 Center for Global Health, The Hospital for Sick Children, Toronto, Canada

## Abstract

**Background:**

Global health education initiatives inconsistently balance trainee growth and benefits to host communities. This report describes a global health elective for medical trainees that focuses on community engagement and participatory research to provide mutually beneficial outcomes for the communities and trainees.

**Methods:**

An eight-year university–community partnership, the Chilcapamba to Montreal Global Health Elective is a two-month shared decision-making research and clinical observership experience in rural Ecuador for medical trainees at McGill University, Canada. Research topics are set by matching community-identified priorities with skillsets and interests of trainees, taking into consideration local potential impact.

**Results:**

Community outcomes included development of a Community Health Worker program, new collaborations with local organizations, community identification of health priorities, and generation of health improvement recommendations. Collaborative academic outputs included multiple bursary awards, conference presentations and published manuscripts.

**Conclusion:**

This medical global health elective engages communities using participatory research to prioritise socially responsible and locally beneficial outcomes.

## Introduction

Medical trainees are increasingly expected to be global citizens and require adequate global health training to meet evolving population demands in this role.^1-6^ Current global health education initiatives offer a mix of both opportunities for personal and professional growth and benefits for the host communities.^2,7-11^ Trainees have shown they can build sustainable relationships in host communities based on trust.^12^ Global health electives should equip trainees to work in culturally diverse and underserved and/or vulnerable populations while fostering values of community service, stewardship of health resources and cultural safety.^1,6,11,13-17^ Cultural safety recognizes power differentials between service providers and recipients, and prioritizes the needs and voice of the recipient.^18^ This differs from “cultural competency,” which assumes the provider has good knowledge about cultural issues, but can lead to “othering” of the receiver as power differentials still continue sometimes unnoticed and unacknowledged.^18,19^

Participatory research involves “systematic inquiry, with the collaboration of those affected by the issue being studied, for purposes of education and taking action or effecting change.”^20^ Participatory research can develop generalizable knowledge, while benefitting the participating community.^21-23^ It can improve research protocols by including local community expertise and supports knowledge translation, social justice and self-determination.^21-23^ It nurtures long term relationships with communities, promotes community capacity to proactively investigate and solve problems and can enable communities to gain control over determinants of their health.^20,22,24^

In this article we describe eight years of experience with a global health elective that benefits both medical trainees and local communities using participatory research for community engagement.

## Methods

### The partner population

The Chilcapamba to Montreal Global Health Elective (CMGHE) takes place in the Canton of Cotacachi (population ~40,000) in the province of Imbabura, Ecuador. It began in Chilcapamba and now involves three additional indigenous communities ranging from 35 to 120 households.

### The elective

The CMGHE is an eight-week research and clinical observership global health elective in rural Ecuador offered to pre-clerkship medical trainees at the end of their first year at McGill University, Canada. The program began in 2008, developed by a McGill medical trainee (AE) and is now overseen by a Spanish speaking multidisciplinary McGill health team working in partnership with local community leaders.

Since 2012, this multidisciplinary McGill team leads a Community Health Worker (CHW) Training Program developed in the same communities. CMGHE trainees work with CHWs as they play a key role in data collection and results dissemination. The CMGHE has been supported by scholarships from the McGill Summer Research Bursary Program.

### The process in Ecuador

The program recruits pre-clinical trainees based on fluency in Spanish and willingness to work in respectful community research partnerships. The program leaders confirm research topics by matching community-identified priorities with trainee skillsets and interests. Trainees finalise a research proposal for submission, after approval of community leaders, to the McGill Bursary Competition and Ethics Committee and the local hospital director in Ecuador.

Pre-departure training by McGill University includes topics related to travel, personal safety, and CMGHE program-specific knowledge, with an emphasis on cultural safety.

Cultural immersion occurs in Ecuador and includes the trainees living with a community family and extensive local social interactions. Data collection occurs in partnership with a community leader and the CHWs.

Trainees are supervised by community leaders including the local project coordinator and have regular contact with a McGill supervisor. Clinical exposure includes daily observerships at the local hospital. The patients observed by trainees excludes study participants, to eliminate conflicts of interest. Trainees participate in clinical observerships most mornings, rotating, based on their interests, between inpatient and outpatient general and subspecialty areas.

At the end of the project, trainees produce a lay summary of preliminary results written in Spanish for community dissemination. McGill requires trainees to provide a written summary and a poster presentation at McGill’s annual Student Research Day. Both the report and poster are reviewed by the community leader to ensure accuracy, respect of culture, and for other active input prior to external dissemination. Local collaborators who made significant contributions are co-authors of all submissions and presentations. Later, a CMGHE trainee or a CHW team member returns to Ecuador to disseminate the final results to community assemblies which is intended to increase knowledge, build capacity and foster community engagement to develop solutions to locally identified challenges.

## Results

The CMGHE has produced research results with multiple academic and community outcomes summarized in Table 1 (Appendix A). Research from this project has documented local prevalence of infectious diseases and parasites; information on sexual health awareness, maternal & child health, intra-familial violence, household food security, and community agricultural practices, which have all increased community knowledge. Total academic outcomes include grant proposals, research bursaries, peer reviewed conference posters and presentations, published manuscripts, and prizes from McGill University for outstanding trainee projects, summarized in Table 1 (Appendix A).

Community outcomes following these results are varied. The early focus groups frequently led to wider discussions around the need for CHWs, which in turn led the McGill team to apply for funding for CHW training. Other outcomes include increased local capacity with community leaders involved in the research projects and now using focus groups to answer their own questions, new links with other local organisations (i.e., to reduce intra-familial violence and improve agricultural practices), increased skills and knowledge of CHWs, and developing new interventions to improve children’s nutrition.

**Figure 1:**
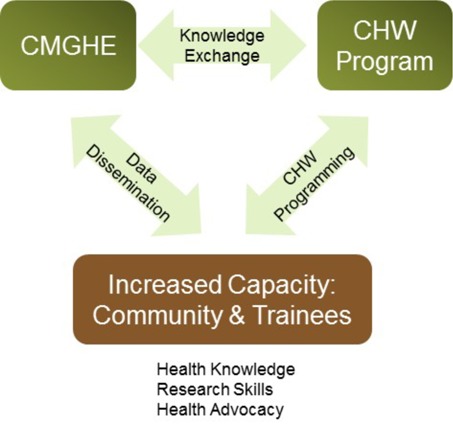
Increased capacity from CMGHE knowledge translation

Figure 1 depicts the flow of knowledge from the CMGHE program towards increased capacity both directly and through the CHW program. Trainees worked closely with CHWs as part of the project setup, execution and data dissemination process. As a result of this collaboration, the CMGHE increased community capacity by empowering CHWs to be more confident health advocates. CHWs have recently independently expanded their role in the communities by offering health messages during community gatherings. CHWs have implemented local health initiatives supported by health knowledge outputs of CMGHE research projects. CHWs have also expanded ownership of their learning by collaborating with the local hospital to address self-identified development needs.

## Discussion

This report describes a structured approach to medical trainee global health education, using the principles of participatory research that benefit the local community, the trainee’s learning, and the larger research community. We believe this approach can offset the ethical and cultural safety dilemmas associated with lack of oversight and mentorship, unbalanced levels of responsibility and unidirectional benefits in favour of the visiting trainee.^26,27^

The participatory research framework to engage community responds to medical trainee interest in developing global health and research skills through cultural immersion and close mentorship. Trainees work closely with CHWs during project setup, execution and dissemination. There is mutual increased capacity for all parties involved as a result of the bidirectional flow of knowledge. In general, CHWs have expressed a greater ability to negotiate with the local hospital due to their gains in health care knowledge since the inception of the CMGHE program. The program is equity oriented with research and health-related learning for both trainees and the community. All trainees and many community members presented their research at academic conferences and were encouraged to develop manuscripts, resulting in publications.^13,25^ The community used the knowledge and was proactive in expanding upon health projects and pursuing new health projects; this is in line with participatory research principles including self-determination and knowledge translation.^22,28^ The participatory research produced its expected ripple effect, with the outcome of one phase becoming the context for the next.^24^ Over the years this generated increasingly complex CMGHE projects which spread to other communities, with similar increased capacity.^24^

Increased capacity occurred as a result of a strong partnership based on trust and mutual respect, essential components of participatory research.^24^ Testimony to the local acceptance of the partnership is the involvement of community members across all projects since 2008, maintaining a sustainable and bilateral relationship between the community and trainees.^24^ The trainee (AE) who initiated the academic community partnership is now a physician actively involved as a CMGHE supervisor and CHW instructor. Other trainees have remained involved with the CMGHE program for years, with some completing a second project, building on previous relationships.

A key limitation of the CMGHE format includes the risks inherent to the acquisition of cultural competence, of “othering” the host.^29^ Other limitations include trainee funding, proficiency in Spanish, and limited time during the two-month elective to grasp the cultural context and refine project tools.

### Conclusion

This medical global health elective engages communities using participatory research. Subsequent studies could evaluate achievement of global health competencies to further validate using participatory research as an approach to global health education for medical trainees.

## Appendix A

**Table 1:** CMGHE research results, community impacts and academic outcomes

Year	Topic	Research Results	Immediate Community Outcomes	Academic Outcomes
2008	Syphilis in prenatal patients	Present, but low prevalence of syphilis in this population	Launch of first focus groups co–led by trainee and community leader Community identifies need for increased health knowledge	Request for CHW program by community McGill Award for best multicultural project Peer-reviewed conference poster and oral presentation Published manuscript (13) 3 McGill Research Bursary Program awards
2009	Intestinal parasites in children	High prevalence of multi-parasitic infections High parasitic infections post treatment	Confirms need for regular deworming treatments through the hospital	2 McGill Research Bursary Program awards Peer-reviewed conference poster
2010	Women’s views on cervical cancer screening	Perceived increase in sexual health knowledge	Community leader participates in mobile clinic for PAP testing	Manuscript submitted and under final review
2011	Access to contraception	Barriers to contraception use	First men’s focus group on contraception	CHW program grant awarded Manuscript submitted and under final review
2012	Access to Maternal & newborn health care Intra-familial violence	Good access to maternal child health care High prevalence of intra-familial violence	New collaboration with local organization to reduce intra-familial violence	2 McGill Research Bursary Program awards Published manuscript (25) 2 peer-reviewed conference posters
2013	Child malnutrition, dietary diversity, household food security	High prevalence of growth stunting, obesity, and food insecurity Opportunity to consume more vegetables	CHWs trained to take anthropometric measurements of children	2 McGill Research Bursary Program awards Manuscript submitted and under review 4 peer-reviewed conference posters
2014	Scale up of 2013 project to new communities Agricultural practices	Similar results to previous year Loss of traditional agricultural practices	Community receives training on agricultural practices and identifies partner NGO: The institute for Self-Reliant Agriculture (Feed the World)	4 McGill Research Bursary Program awards McGill Global Health Program award for best poster Manuscript submitted and under review 2 peer-reviewed conference posters
2015	Child education on nutrition Sourcing of agricultural products	Development of a play for children on healthy eating	CHWs develop community presentations on nutrition	McGill Research Bursary Award 2 peer reviewed conference poster presentations McGill prize in multicultural and international medicine Manuscript in progress
2016	Dental health assessment	Poor dental health High prevalence of dental pain Low use of local dental health service	8 CHWs trained in dental hygiene New collaboration with dental mobile clinics	2 McGill Research Bursary Program awards Manuscript in progress
